# Acupuncture decreases risk of stroke among patients with atrial fibrillation: A nationwide investigation

**DOI:** 10.1097/MD.0000000000031889

**Published:** 2022-12-02

**Authors:** Wei-Syun Hu, Cheng-Li Lin

**Affiliations:** a School of Medicine, College of Medicine, China Medical University, Taichung, Taiwan; b Division of Cardiovascular Medicine, Department of Medicine, China Medical University Hospital, Taichung, Taiwan; c Management Office for Health Data, China Medical University Hospital, Taichung, Taiwan.

**Keywords:** acupuncture, atrial fibrillation, stroke

## Abstract

The authors aim to investigate retrospectively the association between acupuncture and risk of stroke in patients with atrial fibrillation (AF). Using the Taiwan National Health Insurance Research Database, AF patients without any acupuncture treatment record were classified as non-acupuncture cohort and 1:1 matching with acupuncture cohort by age, gender, and all comorbidities. To calculate the risk of stroke in case and control groups, cox proportional hazard models were used and presented by hazard ratios, adjusted hazard ratios (aHRs) and 95% confidence intervals (CIs). Two equally distributed groups of AF individuals with and without acupuncture were included. After adjusting for risk factors, AF subjects with acupuncture conferred a lower risk of stroke (aHR = 0.46, 95% confidence intervals [CI] = 0.38–0.54), ischemic stroke (aHR = 0.47, 95% CI = 0.39–0.56) and hemorrhagic stroke (aHR = 0.35, 95% CI = 0.19–0.67), compared to the controls. AF patients receiving acupuncture was associated with a decreased risk of stroke.

## 1. Introduction

Acupuncture, a traditional herb medicine therapy, is a popular treatment approach in the Chinese population; indeed, several cardiovascular disease modifying effects of acupuncture have been reported.^[1–5]^ Global burden of atrial fibrillation (AF) has been reported, mainly through adverse cerebral and heart diseases.^[6–8]^ AF – all paroxysmal, persistent and chronic subtypes, are known to have an increased risk of stroke.^[9,10]^ To this end, several easy-to-use, validated and well-established tools for risk determination have been made and reported to be of high capacity and reliability.^[11–15]^ The disease modifying approaches would be of interesting in clinical practice concerning the risk estimation of stroke in this AF population. Hence, the authors present the results of a retrospective database analysis of the Taiwan National Health Research Institute and argue whether there is an association between acupuncture use for stroke risk in patients with AF.

## 2. Methods

### 2.1. Data source

We conducted this study by using the Longitudinal Health Insurance Database 2000, which was the subset of NHIRD, and randomly selected 1 million individuals as the sampled patients.^[16–18]^ All diagnoses in the database were coded according to the International Classification of Disease, Ninth Revision, Clinical Modification (ICD-9-CM). The Research Ethics Committee of China Medical University and Hospital in Taiwan approved the study (CMUH-104-REC2-115-CR7).

### 2.2. Study population

We identified acupuncture and non-acupuncture cohort to clarify the association between acupuncture therapies in AF patients and the risk of stroke. The acupuncture cohort was defined as patients firstly received acupuncture treatment after a new diagnosis of AF between January 1, 2000 and December 31, 2012. The acupuncture procedure code included B41, B42, B45, B46, B80-B84, B90-B94, P27041, P31103, P32103, P33031 and electro acupuncture: B43, B44, B86-B89, and P33032. The index date for the patient receiving acupuncture was determined by the date of the initial receiving date of acupuncture. AF patients without any acupuncture treatment record were classified as non-acupuncture cohort and 1:1 matching with acupuncture cohort by age, gender, and all comorbidities. The study period was from the first acupuncture date (index date) to the occurrence date of stroke, withdrawn from NHIRD, death or until December 31, 2013. Patients who were less than 18 years old or having a diagnosis of stroke before acupuncture treatment or ever received anticoagulation therapy were excluded in this study. In this study, we included age, gender and comorbidities, which were important confounding factors. We measured patients with at least 2 claims for outpatient visits or at least 1 claims hospitalization visit at the baseline by the principal and secondary diagnoses for diseases considered possible comorbidities associated with stroke. The comorbidities were defined before index date and contained hypertension, coronary artery disease, peripheral arterial occlusion disease, diabetes mellitus, hyperlipidemia, congestive heart failure, hyperthyroidism, sleep disorder, anxiety, alcoholism, tobacco use, obesity, gout, chronic obstructive pulmonary disease, chronic kidney disease or end stage renal disease, anemia, cancer, and rheumatologic diseases. ICD codes for the diseases are shown in the Supplementary Table, Supplemental Digital Content, http://links.lww.com/MD/H970.

### 2.3. Statistical analysis

We compared the difference of continuous and categorical variable by t-test and chi-square test, respectively, between 2 cohorts. To calculate the risk of stroke in case and control groups, cox proportional hazard models were used and presented by hazard ratios, adjusted hazard ratios (aHRs) and 95% confidence intervals (CIs). We plotted the cumulative incidence curves was by Kaplan–Meier method and the difference of curves assessed by log-rank test. All statistical analyses were performed using SAS statistical software, version 9.4 (SAS Institute Inc, Cary, NC). The figure of cumulative incidence curve was plotted by R software. The significant criteria set up at 2 side *P*-value < .05.

## 3. Results

Of total 3558 eligible study subjects, 1779 patients were with acupuncture treatment after a diagnosis of AF, and the others were AF patients without any medical record of acupuncture treatment. The dominant age group in this study were aged more than 65 years. Patients with acupuncture treatment had 1680 (94.4%) patients coexisting rheumatologic disease, 1379 (77.5%) had hypertension and 1211 (68.1%) had coronary artery disease, which were the most frequent comorbidities in case group. No significant difference was shown between 2 groups (Table 1).

**Table 1:** Characteristics of AF patients according to accept acupuncture.

Variables	Accepted acupuncture				*P*-value
	No (n = 1779)		Yes (n = 1779)		
	n	%	n	%	
**Age group**					.35
18–49	289	16.3	258	14.5	
50–64	546	30.7	563	31.7	
More than 65	944	53.1	958	53.9	
Mean ± SD(a)	64.4(14.7)		64.4(13.9)		.94
**Sex**					
Female	840	47.2	867	48.7	
Male	939	52.8	912	51.3	
**Comorbidity**					
Hypertension	1377	77.4	1379	77.5	.94
Coronary artery disease	1162	65.3	1211	68.1	.08
Peripheral arterial occlusion disease	207	11.6	191	1.7	.39
Diabetes mellitus	354	19.9	335	18.8	.42
Hyperlipidemia	905	50.9	871	49.0	.25
Congestive heart failure	721	40.5	715	4.2	.84
Hyperthyroidism	146	8.21	140	7.87	.71
Sleep disorder	893	50.2	908	51.0	.62
Anxiety	826	46.4	838	47.1	.69
Alcoholism	124	6.97	117	6.58	.64
Tobacco use	41	2.30	37	2.08	.65
Obesity	49	2.75	43	2.42	.53
Gout	497	27.9	489	27.5	.76
COPD	693	39.0	692	38.9	.97
CKD or ESRD	171	9.61	150	8.43	.22
Anemia	276	15.5	289	16.3	.55
Cancer	122	6.86	92	5.17	.03
Rheumatologic disease	1688	94.9	1680	94.4	.55
			

Chi-Square Test.

a *t* test.

AF = atrial fibrillation, CKD = chronic kidney disease, COPD = chronic obstructive pulmonary disease, ESRD = end stage renal disease, SD = standard deviation.

Table 2 presented the number of stroke and hazard ratio between with and without acupuncture treatment among AF patients. Patients with acupuncture treatment had decreased risk (aHR = 0.46, 95% confidence intervals [CI] = 0.38–0.54) of developing stroke compared to the comparison cohort. Other risk factors of stroke included increasing age, female (aHR = 1.22, 95% CI = 1.01–1.47), with hypertension (aHR = 1.52, 95% CI = 1.15–2.01), diabetes (aHR = 1.29, 95% CI = 1.05–1.57), congestive heart failure (aHR = 1.25, 95% CI = 1.05–1.50) and alcoholism (aHR = 1.54, 95% CI = 1.01–2.34) after adjusted for age, gender and comorbidities.

**Table 2:** Cox model with hazard ratios and 95% confidence intervals of stroke associated with accepted acupuncture and covariates among AF patients.

Variable	Stroke	Crude			Adjusted^†^		
	no. (n = 539)	HR	(95% CI)	*P*-value	HR	(95% CI)	*P*-value
**Accepted acupuncture**							
No	292	1.00	Reference		1.00	Reference	
Yes	247	0.50	(0.42, 0.59	<.001	0.46	(0.38, 0.54)	<.001
**Age group**							
18–49	31	1.00	Reference		1.00	Reference	
50–64	146	2.58	(1.75, 3.80)	<.001	2.22	(1.49, 3.32)	<.001
More than 65	362	4.44	(3.08, 6.41)	<.001	3.57	(2.40, 5.32)	<.001
**Sex**							
Female	292	1.28	(1.08, 1.52)	.004	1.22	(1.01, 1.47)	.04
Male	247	1.00	Reference		1.00	Reference	
**Comorbidity (ref = non**-)							
Hypertension	473	2.34	(1.81, 3.03)	<.001	1.52	(1.15, 2.01)	.003
Coronary artery disease	404	1.51	(1.24, 1.84)	<.001	1.10	(0.90,1.36)	.36
Peripheral arterial occlusion disease	68	1.35	(1.04, 1.74)	.02	1.02	(0.78, 1.32)	.91
Diabetes mellitus	142	1.62	(1.34, 1.97)	<.001	1.29	(1.05, 1.57)	.01
Hyperlipidemia	283	1.20	(1.01, 1.42)	.04	0.95	(0.79, 1.14)	.55
Congestive heart failure	261	1.61	(1.36, 1.90)	<.001	1.25	(1.05, 1.50)	.01
Hyperthyroidism	31	0.67	(0.47, 0.96)	.03	0.79	(0.55, 1.14)	.20
Sleep disorder	259	0.99	(0.84, 1.17)	.90	0.90	(0.75, 1.08)	.24
Anxiety	243	0.92	(0.77, 1.09)	.31	0.83	(0.70, 1.00)	.05
Alcoholism	32	1.01	(0.71, 1.44)	.96	1.54	(1.01, 2.34)	.04
Tobacco use	8	0.92	(0.46, 1.85)	.82	0.87	(0.39, 1.95)	.74
Obesity	8	0.62	(0.31, 1.25)	.18	0.78	(0.39, 1.58)	.49
Gout	168	1.26	(1.05, 1.52)	.01	1.12	(0.93, 1.36)	.24
COPD	219	1.24	(1.04, 1.47)	.02	1.00	(0.83, 1.20)	.96
CKD or ESRD	39	1.11	(0.80, 1.54)	.54	1.18	(0.93, 1.49)	.18
Anemia	90	1.24	(0.99, 1.55)	.06	1.18	(0.93, 1.49)	.18
Cancer	16	0.68	(0.41, 1.12)	.13	0.63	(0.38, 1.03)	.07
Rheumatologic disease	519	1.80	(1.15, 2.82)	.01	1.26	(0.80, 1.99)	.32

AF = atrial fibrillation, CI = confidence interval, CKD = chronic kidney disease, COPD = chronic obstructive pulmonary disease, ESRD = end stage renal disease, HR = hazard ratio.

Crude HR represented relative hazard ratio; Adjusted HR^†^ represented adjusted hazard ratio: mutually adjusted for age sex and, comorbidities.

**Figure 1.**
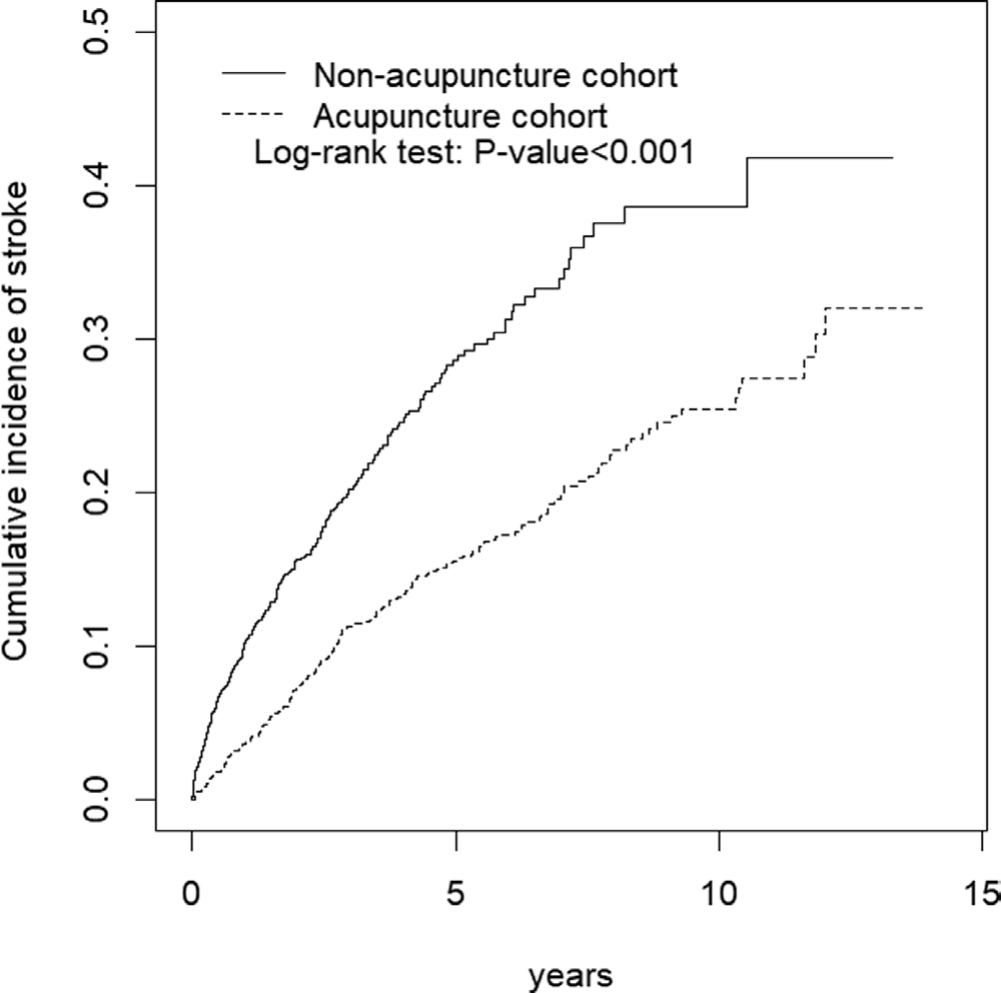
Cumulative incidence of stroke between the acupuncture cohort and the non-acupuncture cohort. The cumulative incidence of stroke in the acupuncture cohort is significantly lower than in the non-acupuncture cohort (log-rank test, *P* < .0001).

Figure 1 demonstrated the cumulative incidence curves of stroke between case and control group, and the cumulative incidence of stroke were significantly lower than non-acupuncture group (*P* < .001).

Table 3 showed the incidence and risk of stroke after stratified with different group. Patients with acupuncture showed lower risk of stroke (aHR = 0.46, 95% CI = 0.38–0.54), ischemic stroke (aHR = 0.47, 95% CI = 0.39–0.56) and hemorrhagic stroke (aHR = 0.35, 95% CI = 0.19–0.67), compared to non-acupuncture treatment cohort. Patients in aged 18 to 49 (aHR = 0.43, 95% CI = 0.20–0.96), 49 to 64 (aHR = 0.42, 95% CI = 0.30–0.59), more than 65 years (aHR = 0.46, 95% CI = 0.37–0.57), female (aHR = 0.37, 95% CI = 0.29–0.47), male (aHR = 0.55, 95% CI = 0.43–0.71) and with comorbidity (aHR = 0.46, 95% CI = 0.39–0.55) showed lower risk of stroke in case group.

**Table 3:** Incidence and Cox proportional hazard regression with hazard ratios and 95% confidence intervals of stroke among AF patients with and without acupuncture stratified by age group, sex, and comorbidity.

Variable	Accepted acupuncture						Crude HR	Adjusted HR‡
	No			Yes				
	Event	Person years	IR†	Event	Person years	IR†		
**Total**	292	3954	73.8	247	7466	33.1	0.50 (0.42, 0.59)	0.46 (0.38, 0.54)
**Ischemic stroke**	267		67.5	230		30.8	0.51 (0.42, 0.61)	0.47 (0.39, 0.56)
**Hemorrhagic stroke**	25		6.32	17		2.28	0.38 (0.20, 0.70)	0.35 (0.19, 0.67)
**Age group**								
18–49	21	866	24.3	10	1314	7.61	0.38 (0.18, 0.81)	0.43 (0.20, 0.96)
49–64	82	1364	60.1	64	2503	25.6	0.46 (0.33, 0.65)	0.42 (0.30, 0.59)
More than 65	189	1725	109.6	173	3649	47.4	0.47 (0.38, 0.58)	0.46 (0.37, 0.57)
**Sex**								
Female	167	1748	95.5	125	3734	33.5	0.39 (0.30, 0.49)	0.37 (0.29, 0.47)
Male	125	2206	56.7	122	3731	32.7	0.64 (0.50, 0.83)	0.55 (0.43, 0.71)
**Comorbidity**								
No	0	8	0.00	0	72	0.00	-	-
Yes	292	3946	74.0	244	7393	33.4	0.50 (0.42, 0.59)	0.46 (0.39, 0.55)

AF = atrial fibrillation, CI = confidence interval, HR = hazard ratio.

† IR, incidence rates, per 1000 person-years.

‡ : represented adjusted hazard ratio: mutually adjusted for, age, sex, and comorbidities.

Table 4 showed the categories of diagnoses among patients who received acupuncture treatment. The most frequent diseases categories were musculoskeletal system and connective tissue (ICD-9-CM: 710–739, 65.8%) and injury and poisoning (ICD-9-CM: 800–999, 57.6%) in this study. Among patients with AF, the risk of stroke exhibited a dose-dependent response with increasing use of acupuncture treatment (Table 5).

**Table 4:** The distribution of acupuncture cohort by disease categories/diagnosis in patients with AF.

Disease (ICD-9-CM)	Acupuncture users	
	(n = 1779)	
	n	%
Infectious and parasitic disease (001–139)	7	0.39
Neoplasms (140–239)	7	0.39
Malignant (140–208)	5	0.28
Benign (210–229)	1	0.06
Endocrine, nutritional and metabolic disease and immunity disorder (240–279)	18	1.01
Blood and blood-forming organs (280–289)	1	0.06
Mental disorder (290–319)	10	0.56
Nervous system (320–389)	123	6.91
Circulatory system (390–459)	63	3.54
Respiratory system (460–519)	38	2.14
Digestive system (520–579)	74	4.16
Genitourinary system (580–629)	23	1.29
Complications of pregnancy, childbirth and the puerperium (630–676)	0	0.00
Skin and subcutaneous tissue (680–709)	7	0.39
Musculoskeletal system and connective tissue (710–739)	1171	65.8
Congenital anomalies (740–759)	13	0.73
Certain conditions originating in the perinatal period (760–779)	0	0.00
Symptoms, signs and ill-defined conditions (780–799)	163	9.16
Injury and poisoning (800–999)	1024	57.6

**Table 5:** Hazard Ratios and 95% confidence intervals of stroke risk associated with cumulative frequency of accepted acupuncture among AF patients.

	Stroke		Hazard Ratio (95% CI)	
	N	No. of Event	Crude	Adjusted^†^
Non-users	1779	292	1 (Reference)	1 (Reference)
Acupuncture users^‡^				
≦6	575	82	0.65 (0.51, 0.83)	0.58 (0.45, 0.75)
7–18	408	54	0.53 (0.39, 0.70)	0.49 (0.45, 0.75)
>18	796	111	0.65 (0.51, 0.83)	0.38 (0.30, 0.48)
*P* for trend			<.001	<.001

CI = confidence interval.

Crude HR represented relative hazard ratio; Adjusted HR† represented adjusted hazard ratio: mutually adjusted for age sex and, comorbidities.

## 4. Discussion

In this study, the authors investigated whether acupuncture could decrease the risk of stroke in Taiwanese patients with AF, using data from the national Taiwan health database with Cox proportional hazard model. Moreover, the decreased risk was more dominant in the subgroup of female gender and hemorrhagic stroke.

The major advantage of this study is that the investigated problem is clearly identified. Moreover, the study methods are clearly explained. Furthermore, the large number of patients included in the analysis is one of the principal strengths of the investigation, making the present research reliable.

The global burden of AF on cardiac prognosis is clear, such as stroke, cardiac failure, thromboembolism disorders and mortality.^[6–10]^ Indeed, several efforts are made for risk stratification of stroke in this population.^[11–15]^ However, compared to risk stratification, it is more important to explore the possible modifying factors between AF and stroke development. Acupuncture, a common therapy in Chinese population, has already been reported to have positive impact on stroke risk modification in cardiac arrhythmia.^[19]^ But to our knowledge, there is no research concerning this issue specifically on AF patients since there is a wide diversity of mechanism of stroke development between AF and non-AF individuals.^[20,21]^ Several hypothetic theories underlying this observation, including anti-inflammatory effect, antioxidant effect, autonomic system modulating effect, rhythm converting effect or comorbidity modifying effects are possible explanations.^[1–5,22–26]^ Nonetheless, the association investigation precludes accurate dedicate biomechanical mechanisms; further large scales bench and clinical studies are awaited.

In this study, acupuncture was shown to reduce stroke risk across all subgroups, especially among female gender and hemorrhagic stroke. Although this might be an interesting observation, such association is probably not causal, but related to comorbidities, medications and other variables that may be difficult to adjust, making a firm conclusion difficult to be made. Whether this observation is a novel finding or just an incidental finding secondary to the limitations of robust adjustment should be taken into account and still needs to be verified in future studies.

## 5. Limitations

Regarding the weaknesses, there is probably a selection bias with using the diagnosis code and selecting patients from a large database. Also, the study is retrospective; therefore, it is unlikely that strict criteria have been followed. As there is no medication record, it cannot be controlled by this. Finally, the reported strength of this association may not be so clinically relevant, despite being original, a clear biological base for this observation is difficult to make.

## 6. Conclusion

We found that acupuncture could decrease risk of stroke development in patients suffering AF.

## Author contributions

Wei-Syun Hu: study concept and design, acquisition of data, analysis and interpretation, manuscript writing, critical revision of the manuscript for important intellectual content and study supervision.

Cheng-Li Lin: acquisition of data, analysis and interpretation.

## Supplementary Material


