# Supplementary material for: Acupuncture decreases risk of stroke among patients with atrial fibrillation: A nationwide investigation
Source: Medicine (Baltimore). 2022 Dec 2;101(48):e31889. doi: 10.1097/MD.0000000000031889 (PMC9726288; doi:10.1097/MD.0000000000031889)
Supplement: Supplementary file 1 [file medi-101-e31889-s001.pdf]

Appendix ICD-9-CM codes for diseases

ICD-9-CM codes	Diagnosis
427.31	Atrial fibrillation
430-437	Stroke
401–405	Hypertension
410-414	Coronary artery disease
440.0, 440.2, 440.3, 440.8, 440.9, 443, 444.0, 444.22, 444.8, 444.9, 447.8, and 447.9	Peripheral arterial occlusion disease
250	Diabetes mellitus
272	Hyperlipidemia
398.91, 402.01, 402.11, 402.91, and 428	Congestive heart failure
242	Hyperthyroidism
307.4 and 780.5	Sleep disorder
300	Anxiety
291, 303, 305, 571.0, 571.1, 571.2, 571.3, 790.3, A215, and V11.3	Alcoholism
305.1	Tobacco use
278	Obesity
274	Gout
491, 492, 496	Chronic obstructive pulmonary disease
585, 586	Chronic kidney disease or end stage renal disease (ESRD)
280-285	Anemia
140-208	Cancer
710, 711, 713, 714, 715, 716, 719, 720, 721, 726, 727, 728, 729, 446	Rheumatologic disease
